# Pharmacy in Georgia

**Published:** 1875-08

**Authors:** 


PHARMACY IN GEORGIA has not hitherto aspired to take position amongst the learned professions. Its votaries have been largely recruited from physicians who were broken down by long years in the saddle; physicians who, skrinking from the hardships of active professional life, had found a more excellent way of accumulating the pecuniary emoluments which they alone valued. For the remainder, the vendors of drugs and medicines in Georgia have been merchants, in a proper sense of the term, and not cultivators of pharmaceutical science and art.

Very recently we find in the newspapers a call made by the druggists of Atlanta, looking to a meeting of the druggists of the State generally, to convene in the city of Macon on Wednesday, the 20th October next, for the purpose of organizing a State Pharmaceutical Association. The call goes on to state: "The first object of said Association will be to establish the relations between druggists, physicians and the people at large, upon just principles, which shall promote the public welfare and tend to mutual strength and advantage." The call is signed by F. King, temporary secretary; Hunt, Rankin & Lamar; Thomas Pullum & Co.; George J. Howard; J. L. & A. J. Pinson; Berry & Co.; Theo. Schumann; Edward Smith, M.D.; W. K. Hodges, M.D.; Harvill & Martin; Fred. King, M.D.; Hurt & Brown; and J. A. Taylor.

Exactly what is designed in establishing, as proposed, relations between the druggists of the State upon the one hand, and physicians and the public upon the other hand, is not explained in the call, and we are therefore left to conjecture for ourselves.

There are various methods by which relationship is established between one class or calling in the business or social community and another. Ames of Massachusetts, and Collins of Connecticut, the one by making a superior shovel at a moderate price, the other by offering a handsome and serviceable axe, have established for themselves relations with dealers in, and consumers of, such commodities in many lands; they have become rich in this world's goods, honored and respected in their several callings. Men of industry and pluck in the mechanic arts, in manufactures, in mercantile pursuits, in the learned professions, in literature, in the fine arts, are continually engaged in the establishment of useful and honorable relations between themselves and the outside world. If the nature of the relations to be established by the Georgia druggists partake of this character; if the establishment of such relations involves, as it ought to do, higher culture and greater efficiency and skill in the members of the proposed Pharmaceutical Association, then we bid them a hearty godspeed in their worthy undertaking. If, upon the other hand, it is proposed to organize a mere commercial league for the pecuniary advantage of its members, it can only result in the failure which generally attends upon such endeavors.

In the interest of the druggists, as well as of the profession and the public, we trust that our former interpretation will prove to be the correct one. We hope to see a Georgia Pharmaceutical Association with a judicious and high-toned code of ethics, laboring in its annual sessions to place its members in a *higher atmosphere* than that of mere trade and merchandise. We hope to see education and professional advancement of its members made its great and leading object; we hope to see charlatanism frowned upon, and a truly professional *esprit de corps* inculcated and encouraged. The druggists of Georgia owe this to themselves; they owe it to their useful and honorable calling; they owe it to the community which supports them; they owe it to the medical profession which encourages and upholds them.

It is perhaps unfortunate that this call emanates alone from the druggists of the city of Atlanta, and, in view of the importance of the object proposed, we would suggest that there is ample time for correspondence and conference with leading druggists all over the State, that the movement may become general and a full attendance secured.

